# Effect of Furocyst on Lipid Profile and Insulin Resistance Across Different Categories of Body Mass Index in Women With Polycystic Ovarian Syndrome (PCOS)

**DOI:** 10.7759/cureus.74571

**Published:** 2024-11-27

**Authors:** Aparna Shukla, Renu Singh, Anuraag Gupta, Apurva Goel, Kiran Tiwari, Satyendra K Singh

**Affiliations:** 1 Centre for Advanced Research, King George's Medical University, Lucknow, IND; 2 Obstetrics and Gynaecology Department, King George's Medical University, Lucknow, IND; 3 Regulatory Department, Chemical Resources (CHERESO), Panchkula, IND; 4 Research and Development Department, Chemical Resources (CHERESO), Panchkula, IND

**Keywords:** cholesterol, fenugreek seeds, metabolic syndrome, obesity, trigonella foenum-graceum

## Abstract

Introduction

Insulin resistance is a fundamental factor in the pathogenesis of polycystic ovarian syndrome (PCOS) and has been found to mediate a close association with obesity and dyslipidemia. While the anti-diabetic and anti-inflammatory properties of fenugreek seed extracts have been demonstrated, research on its anti-hyperlipidemic properties is still in its novice stage, with inconclusive evidence. The present study assessed the impact of fenugreek seed extracts rich in furostanolic saponins (Furocyst) on lipid profiles across different categories of body mass index (BMI) in women with PCOS.

Methodology

The study was a single-blinded, randomized clinical study conducted among 230 patients between 18 and 45 years of age, presenting to the Gynecology and Obstetrics OPD for treatment of PCOS. After screening for eligibility, patients were enrolled and randomized into the experimental group (receiving Furocyst BD for three months) and the placebo group. Blood samples collected before treatment and after the completion of treatment were investigated for insulin resistance and lipid profile. The final analysis was conducted on 188 patients (104 in the Furocyst group and 84 in the placebo group) and stratified for different categories of BMI (based on WHO classification).

Results

A significant reduction in the mean BMI in all patients overall and in patient subgroups according to BMI was noted after 12 weeks of treatment with Furocyst, which was statistically significant in the obese (p<0.001). The HOMA-IR (Homeostatic Model Assessment for Insulin Resistance) index was also reduced in the Furocyst group across all BMI categories, including sub-classes of obese (p<0.001). The lipid-lowering effects of Furocyst were observed on total cholesterol, triglyceride, and VLDL (very-low-density lipoprotein) in all patients, irrespective of the initial BMI category (p<0.05). The drug did not affect the mean serum HDL (high-density lipoprotein) levels. In obese patients, Furocyst also exhibited a statistically significant reduction in LDL-HDL ratio and cholesterol-HDL ratio.

Conclusion

The present study demonstrates the insulin-sensitizing, glucose-regulating, anti-obesity, and anti-hyperlipidemic properties of Furocyst in women with PCOS. The overweight and obese seem to benefit most from the drug. The use of Furocyst may be considered a pragmatic approach to treating PCOS-related symptoms and improving metabolic disturbances, specifically by optimizing the lipid profile in the affected women and lowering cardiovascular risk factors in the long term.

## Introduction

Insulin resistance is a fundamental factor in the pathogenesis of polycystic ovarian syndrome (PCOS) and has been found to mediate a close association with obesity and dyslipidemia [1]. Women with PCOS usually tend to be either overweight or obese [2]. While the causes of PCOS are still not clearly understood, the development of insulin resistance secondary to obesity is strongly correlated. In fact, obesity has been independently associated with insulin resistance, aggravating features of anovulation and hyperandrogenism [3]. While PCOS may develop in women, regardless of physical condition, including both underweight and overweight women, the consistent association between PCOS and obesity most likely explains the various exacerbated metabolic and reproductive irregularities observed in the obese [2].

Besides obesity, PCOS also serves as an important model of lipid alterations that initiate during the adolescent or reproductive period [4]. Based on evidence from prior studies, dyslipidemia is a prevalent metabolic abnormality in young women with PCOS [5], and incidence is reported to be as high as 70% [6]. Women with PCOS and insulin resistance have significantly increased triglyceride (TG), low-density lipoprotein (LDL), apolipoprotein B (Apo-B), TG/high-density lipoprotein (TG/HDL), and Apo-B/Apo-A levels. A considerably decreased HDL and Apo-A levels were found in women with hyperandrogenism. Dyslipidemia in an insulin-resistant state is characterized by elevated plasma levels of cholesterol, LDL, very-low-density lipoproteins (VLDL), TGs, and/or HDL levels in PCOS [7].

Conventional allopathic drugs for the treatment of PCOS to allay menstrual irregularities and induce ovulation have varied response rates with a wide scope of contraindications and a basket of side effects. Recently, to explore more sustainable alternatives, the focus has shifted to safer complementary medicines [8,9]. Emerging evidence indicates that fenugreek seed extracts, by lowering insulin resistance, alleviate the symptoms of PCOS and promote weight loss, with improvements in hormonal profile and ovary-related parameters [10-15]. While the anti-diabetic and anti-inflammatory properties of the formulation have been demonstrated in animal models and humans, research on its anti-hyperlipidemic properties in PCOS is still in its novice stage, with inconclusive evidence [16-18]. The purpose of this study was to assess the impact of fenugreek seed extracts rich in furostanolic saponins (Furocyst) on lipid profiles across different categories of body mass index (BMI) in women with PCOS.

## Materials and methods

Study design

The present study is a single-center, single-blinded, randomized, placebo-controlled clinical trial conducted after approval by the Institutional Ethics Committee (IEC) (no. 208/Ethics/2019 dated 13-03-2019). This trial is registered in the Clinical Trial Registry India (CTRI) with a registration date of 14/02/2020, identification number CTRI/2020/02/023360.

The present research paper is part of a broader study that evaluates the efficacy of Furocyst in alleviating clinical symptoms of PCOS and improving hormonal profile in human participants, as well as exploring mechanisms of its mechanism of action in PCOS mouse models. This paper intends to explore the anti-lipidemic action of fenugreek seed extracts rich in furostanolic saponins (Furocyst) in PCOS patients belonging to different categories of BMI.

Eligibility criteria

To be eligible for inclusion in the study, patients were required to be pre-menopausal, with ages ranging from 18 to 45 years and a diagnosis of PCOS according to the Rotterdam criteria; with adequate hepatic, renal, cardiac, and hematological functions; and with stable weight for the last two months (recall-based). Patients not willing to participate and did not give informed consent to participate in the study; those with acute or chronic medical illness such as hepatic, cardiac, or renal insufficiency, COPD, gastrointestinal disorders, Cushing's syndrome, congenital adrenal hyperplasia, uncontrolled hypertension, smoking, and hypogonadism; using oral contraceptives; smoking or drug-addicted women; pregnant and lactating mothers; with psychiatric illness; and those diagnosed with androgen-secreting tumors were excluded.

Patient enrollment and study procedure

The recruitment and inclusion of participants were conducted at the Obstetrics & Gynaecology Outpatient Department (OPD) of King George's Medical University, Lucknow, India, to investigate the treatment of PCOS. The sample size was calculated by applying the standard formula for randomized controlled trials (RCTs).

$n (per \text{ }study \text{ }group)=((z_{(1-&alpha;/2)} + z_&beta; )^2 . \text{ }2 \text{ }. \text{ }p ̅ (1-p ̅ ) )/(d ̅ )^2$

The trial by Swaroop et al. [14], which evaluated the efficacy of *Trigonella foenum-graecum* seed extract (fenugreek seed extract, Furocyst, two capsules of 500 mg each/day) extraction on the reduction of ovarian volume and the number of ovarian cysts, was referenced for sample size calculation. The study reported that 71% of participants had returned to their regular menstrual cycle upon the completion of the treatment with Furocyst. In our study, anticipating an absolute difference of 20% between the Furocyst group and the placebo group, sample size estimation was done. Considering a confidence limit of 95% and 80% power of study, along with a 20% loss-to-follow-up rate, the minimum desired sample size was 226.

A total of 230 patients meeting the eligibility criteria were enrolled in the study. With an emphasis on crucial document archiving and adherence to international ethical standards as stated in the Declaration of Helsinki and its subsequent amendments, the study was carried out in compliance with the guidelines established by the International Conference on Harmonization (ICH) for Good Clinical Practices (GCP). The patients' understanding of the study goals and procedure and acceptance of their random allocation to either the intervention group or the control group were documented through informed written consent. The consent forms were signed by either the subjects themselves or their legal representatives, and strict measures were implemented to protect patient confidentiality throughout the study.

Randomization, treatment, follow-up, and study compliance

The patients were assigned to either the intervention group (receiving Furocyst) or the control group (receiving placebo) randomly through computer-generated random number tables.

Demographic data, anthropometric measurements, and clinical data (pertaining to presenting complaints, menstrual cycle, and clinical features) were recorded on the first visit. Laboratory investigations were performed to establish baseline measurements, which included hemoglobin, total leukocyte count (TLC), fasting blood sugar, HbA1c, fasting insulin, Homeostatic Model Assessment for Insulin Resistance (HOMA-IR, a proxy measure of insulin resistance), total lipid profile consisting of total cholesterol (CH), HDL, LDL, VLDL, LDL-HDL ratio, CH-HDL ratio, and triglycerides (TG). Hormonal profiling and ultrasonography were also conducted during the second to fifth day of the menstrual cycle.

The participants in the intervention group received the investigational product, formulated as a hard gelatin capsule containing 500 mg of fenugreek seed extract (Furocyst^TM^) in the prescribed dosage of 500 mg/one capsule to be taken twice daily (BD) for 12 weeks. The investigational product was stored at room temperature in a cool and dark environment, ensuring protection from direct sunlight. No adjustments were made to the dosage of concomitant medications.

Those assigned to the control group received a placebo (maize starch) in a similar-looking capsule and were prescribed in a schedule analogous to the intervention tested.

At one time, the patients were given drugs for four weeks only and were asked to bring the empty blister packets on the next visit. Follow-up visits were scheduled every four weeks for comprehensive assessment. A thorough physical examination was conducted during each follow-up visit, and detailed documentation was completed. The next batch of drugs was provided to the participants with the same instructions. The participants in both groups were advised not to change their routine diet or physical activities and to regularly take the drug in the prescribed dose and schedule. Moreover, telephonic reminders were given to all patients to ensure that they attended the follow-up visits on time.

At the end of the 12-week study period, repeat laboratory investigations and ultrasonography (to assess the ovarian volumes) were performed to evaluate changes in various parameters under consideration.

Assessment of safety

For all patients, serum glutamic oxaloacetic transaminase (SGOT), serum glutamic-pyruvic transaminase (SGPT), alkaline phosphatase (ALP), blood urea nitrogen (BUN), and serum creatinine levels were carefully evaluated at baseline and at the end of the 12-week treatment period to assess the comprehensive safety profile of Furocyst. Also, the impact of Furocyst on hemoglobin levels and TLC was analyzed at both the start and the end of the study.

Statistical analysis

IBM SPSS Statistics for Windows, Version 16 (Released 2007; IBM Corp., Armonk, New York) was used for both data entry and analysis. The patients in this study were categorized based on their BMI using the WHO classification system: normal weight: 18.5-24.9 kg/m^2^, overweight: 25-29.9 kg/m^2^, and obese: ≥30 kg/m^2^. Obese class I (BMI 30-34.9 kg/m^2^), class II (BMI 35-39.9 kg/m^2^), and class III (≥40 kg/m^2^) were further classifications for patients with BMIs in the obese range. Using the proper statistical procedures (chi-square test, Mann-Whitney U test), all continuous variables were compared between the study groups at baseline and at the 12-week mark. The paired t-test/Wilcoxon signed-rank test was used for continuous variables, while the McNemar test was used for categorical variables in the before-and-after study. A p-value of <0.05 was considered statistically significant.

## Results

**Figure 1:**
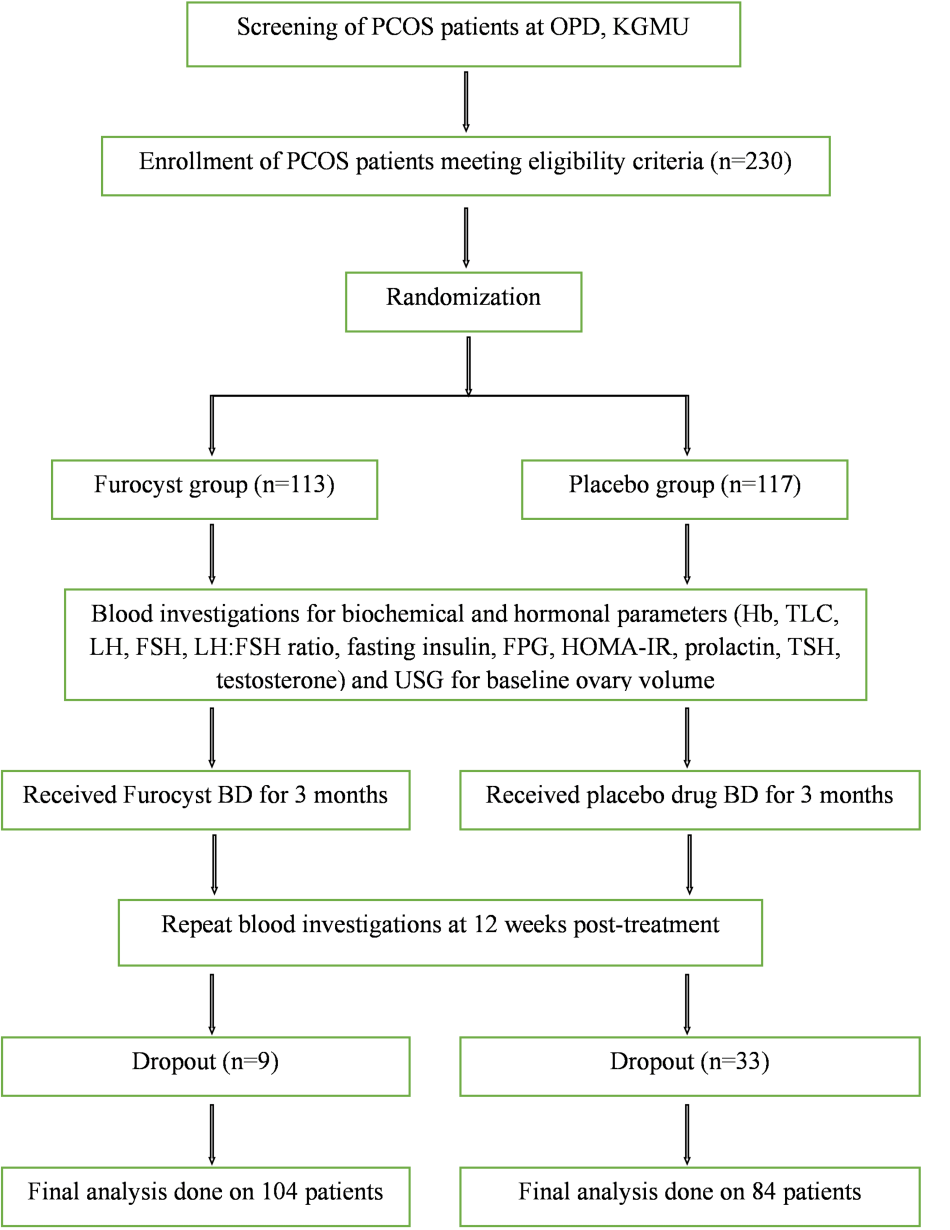
CONSORT diagram CONSORT: consolidated standards of reporting trials

A total of 230 participants were initially recruited for the study, with 113 assigned to the Furocyst group and 117 to the placebo group. However, during the course of the study, 42 participants dropped out, with nine from the Furocyst group and 33 from the placebo group. Therefore, the final analysis included data from 188 patients, with 104 in the Furocyst group and 84 in the placebo group (Figure 1).

Baseline characteristics

Among the 188 study participants, 30.3% were overweight, and 38.3% were classified as obese. The distribution of participants between the Furocyst and placebo groups according to BMI categories at the time of enrollment was statistically comparable (p=0.625) (Table 1).

**Table 1:** Distribution of the study participants according to BMI categories at baseline in both study groups * The p-value was based on the chi-square test, and p<0.05 was considered statistically significant. BMI: body mass index

BMI Category	Placebo (n=84)		Furocyst (n=104)		Chi-Square Value	p-value
	Frequency	Percentage	Frequency	Percentage		
Normal	24	28.6	35	33.7	2.609	0.625
Overweight	29	34.5	28	26.9		
Obese class I	13	15.5	19	18.3		
Obese class II	12	14.3	11	10.6		
Obese class III	6	7.1	11	10.6		

The baseline demographic characteristics and laboratory parameters of patients with BMIs in the normal range, overweight, and obese were also comparable between the two study groups (Table 2).

**Table 2:** Comparison of age, fasting blood sugar, insulin resistance, and lipid profile at baseline in the three BMI categories between the study groups * The p-value was based on the Mann-Whitney U test, and p<0.05 was considered statistically significant. The data were represented as mean±SD. All parameters measured at baseline and stratified according to different BMI categories (normal, overweight, and obesity) were comparable between the placebo and Furocyst groups (p>0.05). BMI: body mass index; FBS: fasting blood sugar; HOMA-IR: Homeostasis Model Assessment of Insulin Resistance; CH: cholesterol; TG: triglyceride; HDL: high-density lipoprotein; LDL: low-density lipoprotein; VLDL: very-low-density lipoprotein

Parameters	Normal		Overweight		Obesity	
	Placebo	Furocyst	Placebo	Furocyst	Placebo	Furocyst
Age	25.38±4.31	25.06±4.89	24.21±3.59	26.75±5.73	24.74±4.30	24.12±3.95
BMI	23.29±1.52	22.32±1.96	28.05±1.39	27.38±1.34	36.66±5.55	36.87±5.67
FBS	101.13±16.62	99.92±15.02	110.59±16.75	101.56±12.82	103.9±15	103.51±17.27
HOMA-IR	3.45±1.82	3.44±1.47	3.23±1.01	3.4±1.35	4.02±1.68	3.69±1.85
CH	174.3±53.9	162.21±50.18	161.23±45.48	168.18±38.93	174.49±50.3	178.95±47.15
TG	176.55±70.25	174.39±60.39	176.21±65.45	172.3±48.03	174.81±64.21	179.48±55.1
HDL	46.32±8.31	43.28±9.3	46.81±7.25	47.78±9.15	48.44±9.36	46.34±9.47
LDL	92.67±49.91	84.06±42.81	79.18±39.02	85.94±39.15	91.09±45.44	96.71±45.28
VLDL	35.31±14.05	34.88±12.08	35.24±13.09	34.46±9.61	34.96±12.84	35.9±11.02
LDL-HDL ratio	2.08±1.26	1.98±0.92	1.72±0.86	1.91±0.99	1.91±0.97	2.2±1.22
CH-HDL ratio	3.85±1.32	3.8±0.99	3.49±0.99	3.67±1.16	3.65±0.97	4.03±1.41

BMI

While BMI did not change significantly in the placebo group (Tables 3-5), we observed a reduction in the mean BMI in all patients overall (Table 3) as well as in patient subgroups according to BMI after the 12-week treatment with Furocyst, which was statistically significant in the obese (p<0.001) (Table 6) and resulted cumulatively from a reduction in the mean BMI across patients in obese class I (p=0.033), obese class II (p=0.001), and obese class III (p=0.004) (Table 7).

**Table 3:** Comparison of BMI at baseline and at 12 weeks between the two study groups * The p-value was based on the Wilcoxon signed-rank test, and p<0.05 was considered statistically significant. BMI: body mass index

BMI	Baseline Value		Final Value		t-statistic	p-value
	Mean	SD	Mean	SD		
Placebo group	29.9	6.6	29.3	6.9	0.834	0.092
Furocyst group	29.4	7.4	28.1	6.5	3.797	<0.001*

**Table 4:** Comparison of changes in BMI, fasting blood sugar, insulin resistance, and lipid profile after 12 weeks of drug treatment in the BMI categories in the placebo group * The p-value was based on the Wilcoxon signed-rank test, and p<0.05 was considered statistically significant. BMI: body mass index; FBS: fasting blood sugar; HOMA-IR: Homeostasis Model Assessment of Insulin Resistance; CH: cholesterol; TG: triglyceride; HDL: high-density lipoprotein; LDL: low-density lipoprotein; VLDL: very-low-density lipoprotein

BMI Category	Parameters	Baseline		At 12 Weeks		Mean Difference	t-statistic	p-value
		Mean	SD	Mean	SD			
Normal (n=24)	BMI	23.30	1.52	23.48	1.95	-0.19	-0.615	0.544
	FBS	101.12	16.62	107.17	14.13	-6.04	-2.247	0.035*
	HOMA-IR	3.45	1.82	3.79	2.00	-0.34	-1.857	0.076
	CH	174.30	53.90	180.63	49.60	-6.33	-0.919	0.368
	TG	176.55	70.25	181.29	62.61	-4.74	-0.918	0.368
	HDL	46.32	8.31	44.09	13.13	2.23	0.870	0.394
	LDL	92.67	49.91	100.28	50.33	-7.61	-0.975	0.340
	VLDL	35.31	14.05	36.26	12.52	-0.95	-0.918	0.368
	LDL-HDL ratio	2.08	1.26	2.58	1.61	-0.50	-1.932	0.066
	CH-HDL ratio	3.85	1.32	4.47	1.79	-0.62	-2.000	0.057
Overweight (n=29)	BMI	28.05	1.39	27.26	2.72	0.79	1.985	0.057
	FBS	110.59	16.75	115.66	15.82	-5.07	-2.326	0.027*
	HOMA-IR	3.23	1.01	3.27	1.27	-0.04	-0.288	0.775
	CH	161.23	45.48	172.59	43.69	-11.36	-2.064	0.048
	TG	176.21	65.45	182.84	58.76	-6.63	-1.107	0.278
	HDL	46.81	7.25	41.24	11.06	5.58	3.265	0.003*
	LDL	79.18	39.02	94.78	43.94	-15.61	-2.460	0.020*
	VLDL	35.24	13.09	36.57	11.75	-1.33	-1.107	0.278
	LDL-HDL ratio	1.72	0.86	2.59	1.69	-0.87	-3.151	0.004*
	CH-HDL ratio	3.49	0.99	4.52	1.85	-1.04	-3.273	0.003*
Obese (n=31)	BMI	36.66	5.55	36.75	5.74	-0.09	-0.231	0.819
	FBS	103.90	15.00	108.77	15.23	-4.87	-2.403	0.023*
	HOMA-IR	4.02	1.68	3.57	1.52	0.45	1.294	0.205
	CH	174.49	50.30	188.44	54.96	-13.94	-2.763	0.010*
	TG	174.81	64.21	172.95	62.06	1.86	0.263	0.794
	HDL	48.44	9.36	43.59	13.50	4.85	1.841	0.076
	LDL	91.09	45.44	110.25	56.07	-19.16	-3.339	0.002*
	VLDL	34.96	12.84	34.59	12.41	0.37	0.263	0.794
	LDL-HDL ratio	1.91	0.97	3.11	2.54	-1.20	-3.175	0.003*
	CH-HDL ratio	3.65	0.97	4.99	2.88	-1.35	-3.116	0.004*

**Table 5:** Comparison of changes in BMI, fasting blood sugar, insulin resistance, and lipid profile after 12 weeks of drug treatment in the sub-classes of the obese category in the placebo group * The p-value was based on the Wilcoxon signed-rank test, and p<0.05 was considered statistically significant. BMI: body mass index; FBS: fasting blood sugar; HOMA-IR: Homeostasis Model Assessment of Insulin Resistance; CH: cholesterol; TG: triglyceride; HDL: high-density lipoprotein; LDL: low-density lipoprotein; VLDL: very-low-density lipoprotein

BMI Category	Parameters	Baseline		At 12 Weeks		Mean Difference	t-statistic	p-value
		Mean	SD	Mean	SD			
Obese class I (n=13)	BMI	32.11	1.53	32.75	2.22	-0.64	-1.506	0.158
	FBS	105.54	12.80	112.46	13.32	-6.92	-1.853	0.089
	HOMA-IR	4.39	2.23	3.41	1.88	0.98	1.410	0.184
	CH	172.87	49.15	197.96	57.03	-25.09	-3.735	0.003*
	TG	173.77	52.56	184.14	51.31	-10.37	-0.667	0.517
	HDL	45.20	10.31	42.53	15.73	2.67	0.652	0.527
	LDL	92.92	50.48	118.60	66.21	-25.69	-3.060	0.010*
	VLDL	34.75	10.51	36.83	10.26	-2.07	-0.667	0.517
	LDL-HDL ratio	2.18	1.26	3.72	3.35	-1.54	-2.112	0.056
	CH-HDL ratio	3.97	1.27	5.68	3.66	-1.71	-2.122	0.055
Obese class II (n=12)	BMI	36.94	1.44	36.39	2.78	0.55	0.824	0.427
	FBS	101.42	14.31	104.92	15.35	-3.50	-1.477	0.168
	HOMA-IR	3.58	0.97	3.60	1.30	-0.02	-0.083	0.935
	CH	178.58	60.51	189.83	57.74	-11.25	-1.478	0.168
	TG	181.50	79.99	174.96	73.80	6.54	1.141	0.278
	HDL	50.92	9.26	44.36	10.90	6.56	1.654	0.126
	LDL	91.37	50.40	110.49	53.96	-19.12	-2.178	0.052
	VLDL	36.30	16.00	34.99	14.76	1.31	1.141	0.278
	LDL-HDL ratio	1.71	0.75	2.75	1.70	-1.04	-2.517	0.029*
	CH-HDL ratio	3.44	0.67	4.63	2.14	-1.19	-2.320	0.041*
Obese class III (n=6)	BMI	45.96	4.17	46.12	4.69	-0.17	-0.132	0.900
	FBS	105.33	21.96	108.50	19.40	-3.17	-0.610	0.569
	HOMA-IR	4.08	1.45	3.84	1.21	0.24	0.277	0.793
	CH	169.83	35.44	165.00	45.68	4.83	0.368	0.728
	TG	163.67	61.42	144.67	59.23	19.00	4.478	0.007*
	HDL	50.50	5.89	44.37	15.28	6.13	0.828	0.445
	LDL	86.60	25.59	91.70	36.65	-5.10	-0.318	0.763
	VLDL	32.73	12.28	28.93	11.85	3.80	4.478	0.007*
	LDL-HDL ratio	1.72	0.49	2.52	0.92	-0.80	-0.869	0.424
	CH-HDL ratio	3.37	0.56	4.23	2.28	-0.87	-0.814	0.453

**Table 6:** Comparison of changes in BMI, fasting blood sugar, insulin resistance, and lipid profile after 12 weeks of drug treatment in the BMI categories in the Furocyst group * The p-value was based on the Wilcoxon signed-rank test, and p<0.05 was considered statistically significant. BMI: body mass index; FBS: fasting blood sugar; HOMA-IR: Homeostasis Model Assessment of Insulin Resistance; CH: cholesterol; TG: triglyceride; HDL: high-density lipoprotein; LDL: low-density lipoprotein; VLDL: very-low-density lipoprotein

BMI Category	Parameters	Baseline		At 12 Weeks		Mean Difference	t-statistic	p-value
		Mean	SD	Mean	SD			
Normal (n=35)	BMI	22.32	1.96	22.53	2.23	-0.21	-0.586	0.562
	FBS	99.92	15.02	91.57	9.18	8.35	3.391	0.002*
	HOMA-IR	3.44	1.47	2.39	1.00	1.06	5.027	0.000*
	CH	162.21	50.18	143.15	34.57	19.06	3.367	0.002*
	TG	174.39	60.39	149.46	48.27	24.93	4.291	0.000*
	HDL	43.28	9.30	44.16	11.05	-0.88	-0.690	0.495
	LDL	84.06	42.81	75.10	34.13	8.96	1.449	0.156
	VLDL	34.88	12.08	29.89	9.65	4.99	4.291	0.000*
	LDL-HDL ratio	1.98	0.92	1.78	0.91	0.19	1.170	0.250
	CH-HDL ratio	3.80	0.99	3.38	0.99	0.41	2.230	0.032*
Overweight (n=28)	BMI	27.38	1.33	26.61	3.24	0.77	1.443	0.161
	FBS	101.56	12.82	91.21	10.05	10.34	4.197	0.000*
	HOMA-IR	3.40	1.35	2.22	1.08	1.18	4.749	0.000*
	CH	168.18	38.93	144.05	30.33	24.14	5.039	0.000*
	TG	172.30	48.03	156.32	41.84	15.98	3.538	0.001*
	HDL	47.78	9.15	45.23	11.64	2.54	1.632	0.114
	LDL	85.94	39.15	75.05	25.04	10.89	2.071	0.048*
	VLDL	34.46	9.61	31.26	8.37	3.20	3.538	0.001*
	LDL-HDL ratio	1.91	0.99	1.74	0.68	0.17	1.240	0.226
	CH-HDL ratio	3.67	1.16	3.34	0.92	0.32	-2.050	0.050
Obese (n=41)	BMI	36.87	5.67	34.60	5.17	2.27	5.274	0.000*
	FBS	103.51	17.27	93.17	11.74	10.34	5.348	0.000*
	HOMA-IR	3.69	1.85	2.21	0.70	1.48	6.015	0.000*
	CH	178.95	47.15	152.04	38.69	26.91	6.369	0.000*
	TG	179.48	55.10	160.15	44.00	19.33	3.470	0.001*
	HDL	46.34	9.47	47.63	10.38	-1.29	1.991	0.183
	LDL	96.71	45.28	82.38	40.40	14.33	2.891	0.006*
	VLDL	35.90	11.02	32.03	8.80	3.87	3.470	0.001*
	LDL-HDL ratio	2.20	1.22	1.80	0.94	0.40	2.918	0.006*
	CH-HDL ratio	4.03	1.41	3.33	1.09	0.69	4.829	<0.001*

**Table 7:** Comparison of changes in BMI, fasting blood sugar, insulin resistance, and lipid profile after 12 weeks of drug treatment in the sub-classes of the obese category in the Furocyst group * The p-value was based on the Wilcoxon signed-rank test, and p<0.05 was considered statistically significant. BMI: body mass index; FBS: fasting blood sugar; HOMA-IR: Homeostasis Model Assessment of Insulin Resistance; CH: cholesterol; TG: triglyceride; HDL: high-density lipoprotein; LDL: low-density lipoprotein; VLDL: very-low-density lipoprotein

BMI Category	Parameters	Baseline		At 12 Weeks		Mean Difference	t-statistic	p-value
		Mean	SD	Mean	SD			
Obese class I (n=19)	BMI	32.02	1.30	30.68	2.82	1.34	2.310	0.033*
	FBS	102.86	12.60	91.68	10.20	11.18	5.172	0.000*
	HOMA-IR	3.63	1.99	2.20	0.85	1.42	3.872	0.001*
	CH	169.94	43.86	141.61	36.64	28.33	4.582	0.000*
	TG	177.13	57.61	159.53	46.51	17.61	2.419	0.026*
	HDL	45.99	10.25	49.69	10.26	-3.70	-2.936	0.009*
	LDL	88.52	44.99	72.11	41.65	16.41	2.105	0.050
	VLDL	35.43	11.52	31.91	9.30	3.52	2.419	0.026*
	LDL-HDL ratio	2.05	1.27	1.48	0.89	0.57	2.367	0.029*
	CH-HDL ratio	3.90	1.47	2.95	0.93	0.95	3.704	0.002*
Obese class II (n=11)	BMI	37.38	1.30	35.05	1.93	2.33	4.472	0.001*
	FBS	106.95	28.19	92.45	15.78	14.49	2.771	0.020*
	HOMA-IR	4.02	2.02	2.19	0.51	1.83	3.123	0.011*
	CH	198.45	46.79	177.00	36.68	21.45	2.120	0.060
	TG	175.18	43.03	166.82	41.72	8.36	1.045	0.320
	HDL	49.46	9.48	48.18	11.89	1.28	0.772	0.458
	LDL	113.95	44.52	105.45	37.70	8.50	0.728	0.484
	VLDL	35.04	8.61	33.36	8.34	1.67	1.045	0.320
	LDL-HDL ratio	2.40	1.14	2.25	0.79	0.14	0.556	0.590
	CH-HDL ratio	4.14	1.30	3.83	0.99	0.32	1.375	0.199
Obese class III (n=11)	BMI	44.75	3.24	40.92	3.93	3.83	3.718	0.004*
	FBS	101.18	9.65	96.45	9.92	4.73	1.592	0.143
	HOMA-IR	3.45	1.54	2.23	0.62	1.22	3.574	0.005*
	CH	175.00	51.49	145.11	35.78	29.89	4.699	0.001*
	TG	187.82	64.90	154.55	45.01	33.27	2.342	0.041*
	HDL	43.84	7.86	43.52	8.52	0.32	0.165	0.872
	LDL	93.60	45.93	77.05	34.18	16.55	2.752	0.020*
	VLDL	37.56	12.98	30.91	9.00	6.65	2.342	0.041*
	LDL-HDL ratio	2.25	1.28	1.76	1.90	0.35	2.746	0.021*
	CH-HDL ratio	4.13	1.51	3.51	1.28	0.62	4.072	0.002*

Insulin resistance

The HOMA-IR index was significantly reduced in the Furocyst group across all BMI categories, including sub-classes of obese (p<0.001). Remarkable improvements in insulin resistance correspondingly reflected a reduction in fasting blood sugar levels in all the categories in the patients receiving Furocyst as against those on placebo (Tables 4-7).

Lipid profile

There was a significant reduction in the mean serum levels of CH, TG, and VLDL in all patients receiving Furocyst, irrespective of the initial BMI category (p<0.05). Although a drop in serum LDL level was noted in the Furocyst group at 12 weeks compared to baseline in patients with normal BMI, overweight, and obese, the decrease was statistically significant only in overweight and obese patients receiving Furocyst. However, the mean serum HDL values did not demonstrate any significant increase post-treatment in this study group. In the obese patients, Furocyst also exhibited a statistically significant reduction in the LDL-HDL ratio and cholesterol-HDL ratio (Table 6).

On stratified analysis of lipid profile among obese sub-classes, interestingly, Furocyst failed to demonstrate improvements in lipid-related parameters in obese class II patients only. Except for changes in the mean LDL values in obese class I and the mean HDL values in obese class III, all other lipid-related parameters changed favorably post-treatment with Furocyst (Table 7).

## Discussion

Metabolic derangements such as insulin resistance and dyslipidemia are commonly associated in patients with PCOS. While insulin resistance has obvious manifestations in overweight and obese women, worsening of insulin resistance has been explicated to be attributed to aberrant fat distribution as women with PCOS gain weight [1,2].

In the present placebo-controlled open-label randomized study, we examined the effect of Furocyst on insulin resistance and lipid profile in normal, overweight, and obese women with confirmed PCOS. After 12 weeks of treatment, a significant weight reduction and consequent reduction in BMI were observed in obese patients receiving Furocyst. The weight-regulating and anti-obesity properties of fenugreek seed extracts, ascribed to the essential basket of phytochemical constituents [19], have been proven in adipocyte cell cultures [20] and animal studies on rat models [17]. Based on our observations, in the absence of lifestyle modification or intake of any other concomitant anti-obesity drugs, the reduction of BMI among patients receiving Furocyst can be hypothesized to be due to the therapeutic effect of furostanolic saponins present in the formulation used in our study.

The TG/HDL and Apo-B/Apo-A ratios are associated with some characteristics of PCOS, such as insulin resistance and obesity [21]. Women with PCOS receiving Furocyst for 12 weeks showed a significant decline in insulin resistance (as assessed by the HOMA-IR index) irrespective of their initial BMI status, with maximum reduction among the obese. The present study invariably reiterated the insulin-sensitizing activity of fenugreek, which has been previously claimed in animal models [22] and human studies [12-14]. Fenugreek seed extracts, particularly concentrated with furostanolic saponins, have been previously reported to ameliorate insulin resistance in mouse models [23].

To date, there is limited and inconsistent clinical data on the anti-hyperlipidemic properties of fenugreek. While some studies reported that fenugreek improved TG [24-26], HDL-c [24,26,27], CH, and LDL-c [25], others found no benefit [28,29]. However, all these studies were conducted on varied populations, some on general patients [25], diabetic patients [26], or postmenopausal women [29]. Recent trials have explored the potential of Furocyst in ameliorating symptoms of PCOS, inducing ovulation, and improving fertility [12-14]. Metabolic derangements from insulin resistance and weight gain have also been proven to result in aberrations in lipid profile parameters. The present trial, for the first time, attempted to demonstrate the effect of Furocyst in regulating lipid levels in women with PCOS.

We observed that Furocyst effectively lowered the serum concentration of CH, TG, and VLDL, with no impact on HDL levels in all participants. Interestingly, the drug seemed to be effective in improving LDL levels in the overweight and obese. Maximum reductions in CH and LDL were observed among the obese, followed by the overweight patients in the Furocyst group. Chevassus et al., in their study on healthy overweight volunteers, reported that repeated administration of fenugreek seed extracts led to a slight but significant decrease in dietary fat consumption without any change in appetite or satiety scores and oxidative parameters [28].

The obese patients also had a significant decline in the LDL-HDL ratio and cholesterol-HDL ratio. These lipoprotein ratios or atherogenic indices are powerful predictors of cardiovascular risk, and the present study paved the way for future long-term trials to explore the cardioprotective role of Furocyst in obese patients in different study populations.

While analyzing the changes in the lipid profile parameters, we could not find any plausible explanation for the failure to elicit significant responses in class II obese patients. In contrast, patients with class I and class III obesity showed excellent improvements in the same. The number of study participants in these sub-groups would have probably been insufficient to show significant differences in these parameters.

It should be emphasized that our study represents a prospective evaluation of a cohort of women with PCOS for a short treatment period of 12 weeks, which is also a major limitation of this study. Also, there was a significant attrition rate, more so in the placebo group. Ideally, further studies involving testing of the effects of the drugs in patients categorically with and without hyperinsulinemia and dyslipidemia and for a longer treatment and follow-up duration are recommended.

## Conclusions

The present study demonstrated the insulin-sensitizing, glucose-regulating, anti-obesity, and anti-hyperlipidemic properties of Furocyst in women with PCOS, and the overweight and obese seemed to be most benefitted from the drug. Studies to explore the various mechanisms through which Furocyst exerts these therapeutic roles are still underway. Nevertheless, the use of Furocyst may be considered a pragmatic approach to treat PCOS-related symptoms and improve metabolic disturbances, specifically by optimizing the lipid profile in the affected women and lowering cardiovascular risk factors in the long term.
